# Supplementary material for: Emulating Artificial Synaptic Plasticity Characteristics from SiO2-Based Conductive Bridge Memories with Pt Nanoparticles
Source: Micromachines (Basel). 2021 Mar 15;12(3):306. doi: 10.3390/mi12030306 (PMC7999862; doi:10.3390/mi12030306)
Supplement: Supplementary file 1 [file micromachines-12-00306-s001.pdf]

micromachines

MDPI

Supplementary Materials

Emulating Artificial Synaptic Plasticity Characteristics from Sio2-based Conductive Bridge Memories with Pt Nanoparticles

Panagiotis Bousoulas [1,*], Charalampos Papakonstantinopoulos [1], Stavros Kitsios [1], Konstantinos Moustakas [1], Georgios Sirakoulis [2] and Dimitris Tsoukalas [1]

[1] Department of Applied Physics, National Technical University of Athens, Iroon Polytechniou 9 Zografou, 15780 Athens, Greece; panbous@mail.ntua.gr (P.B.); babispapak@gmail.com (C.P.); stkitsio@mail.ntua.gr (S.K.); Konstantinos_mous@hotmail.com (K.M.); dtsouk@central.ntua.gr (D.T.)

[2] Department of Electrical and Computer Engineering, Democritus University of Thrace, Xanthi 67100, Greece gsirak@ee.duth.gr (G.S.)

* Correspondence: panbous@mail.ntua.gr; Tel.: +302107721679

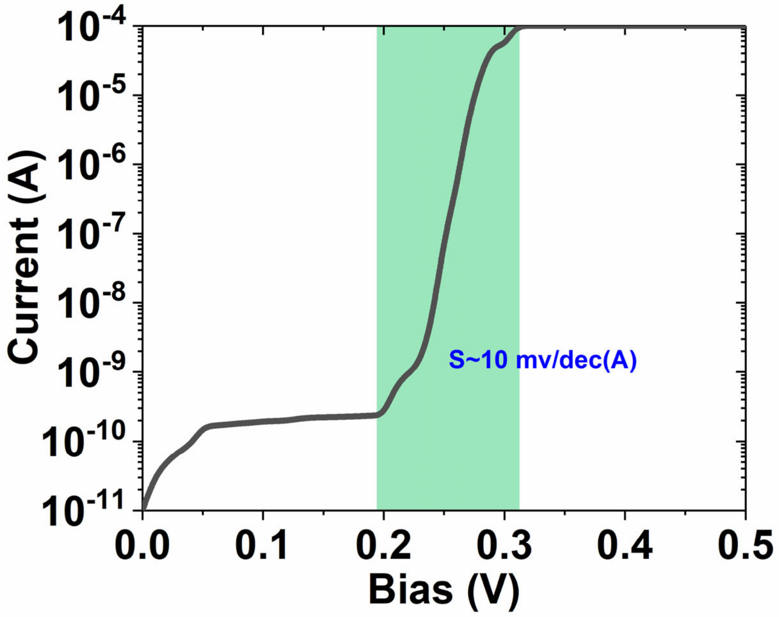

Figure S1. Distribution of the transition slope during the execution of the first switching cycle.

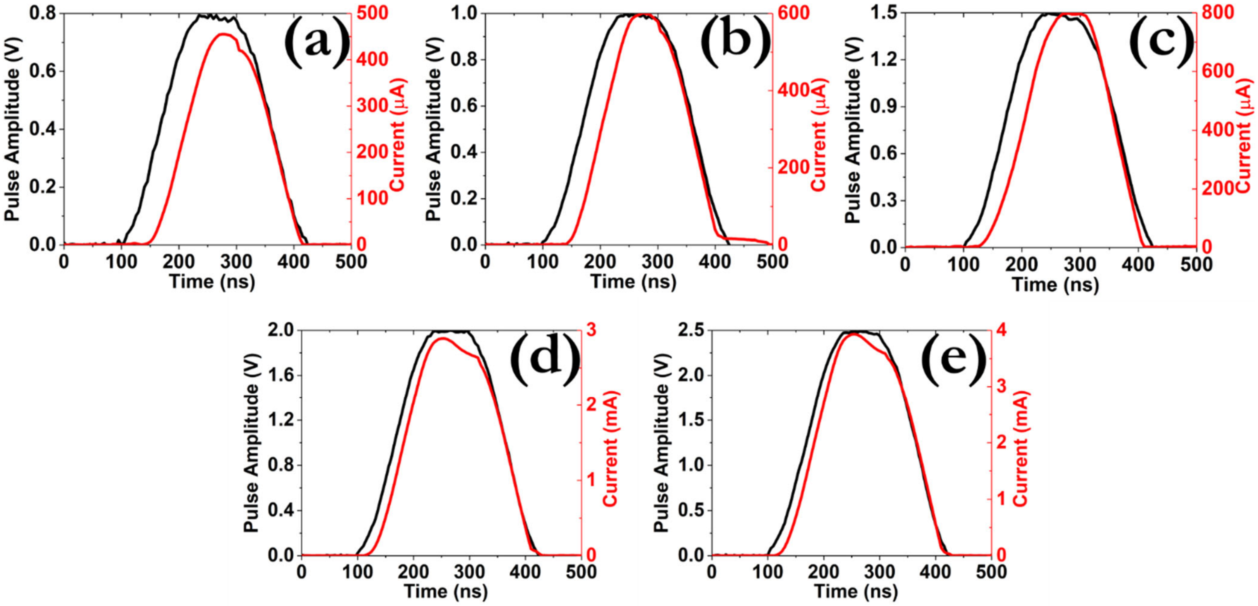

Figure S2. Measured transient current responses (red line) as a function of the pulse amplitude (black line) during the enforcement of square pulses of fixed 100 ns width and amplitudes of (a) 0.8 V, (b) 1 V, (c) 1.5 V, (d) 2 V and (e) 2.5 V, respectively.

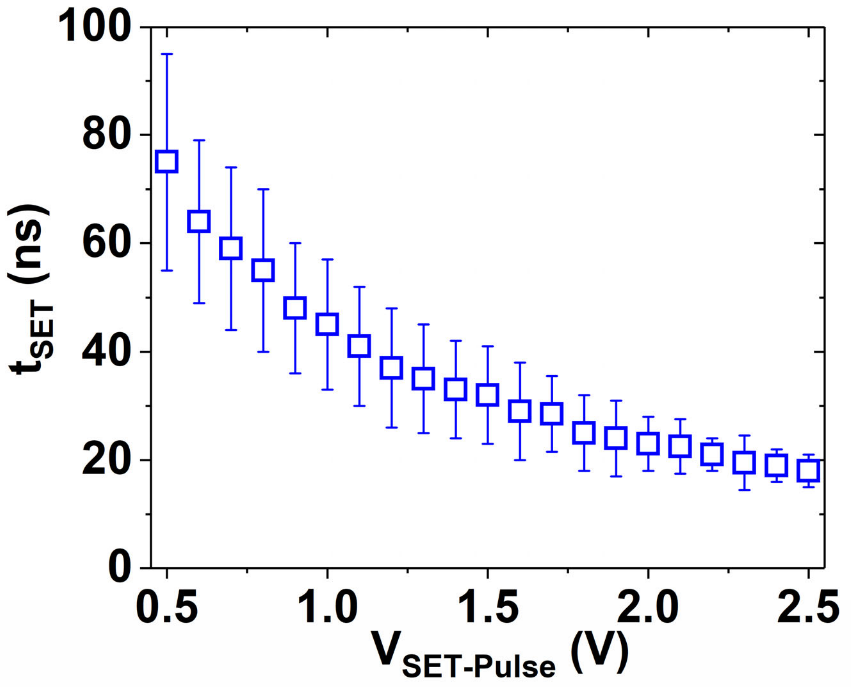

Figure S3. Distribution of the SET process switching time (t_{SET}) as a function of the applied pulse amplitude.

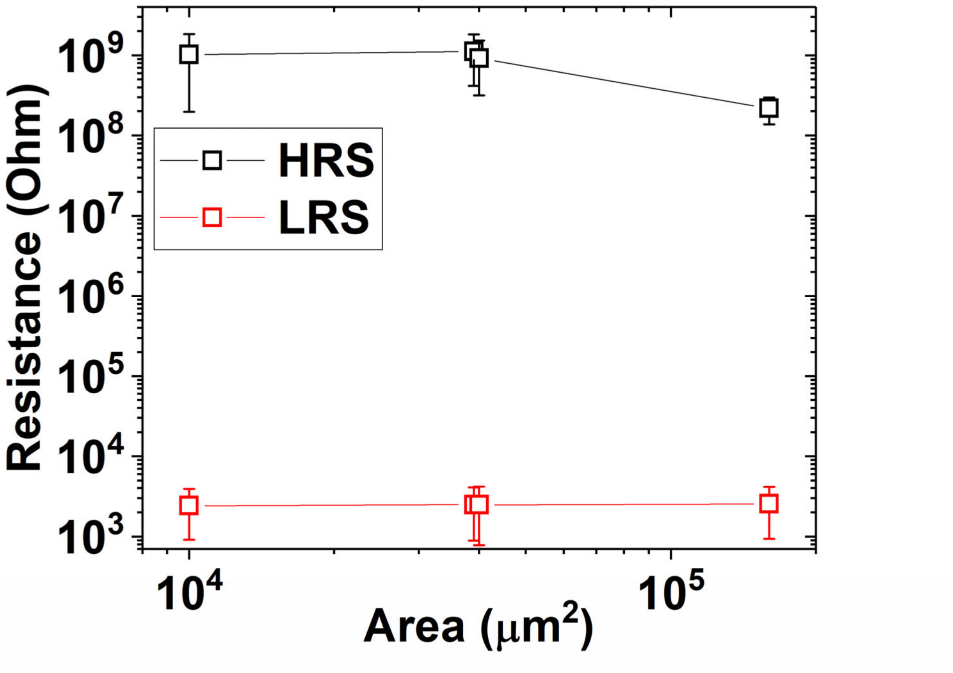

Figure 4. Area dependence of the resistance states for both HRS and LRS (read voltage 100 mV).

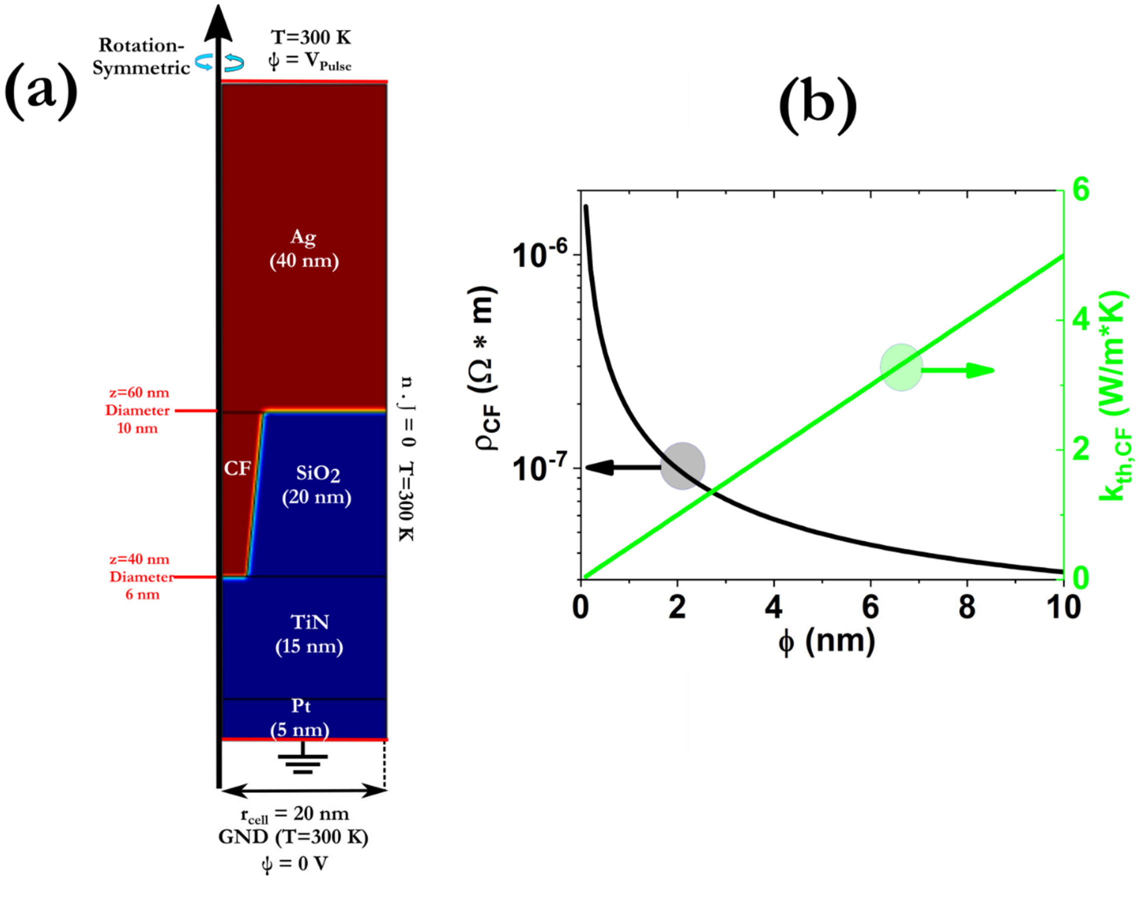

Figure S5. (a) Cross-section representation of the simulated memory cell in 2D axisymmetric geometry with the respective boundary conditions. The red color signifies the distribution of the CF's diameter that consist of either Ag NCs, (b) distribution of the electrical resistivity and thermal conductivity as a function of the effective CF diameter φ for Ag-based CFs.

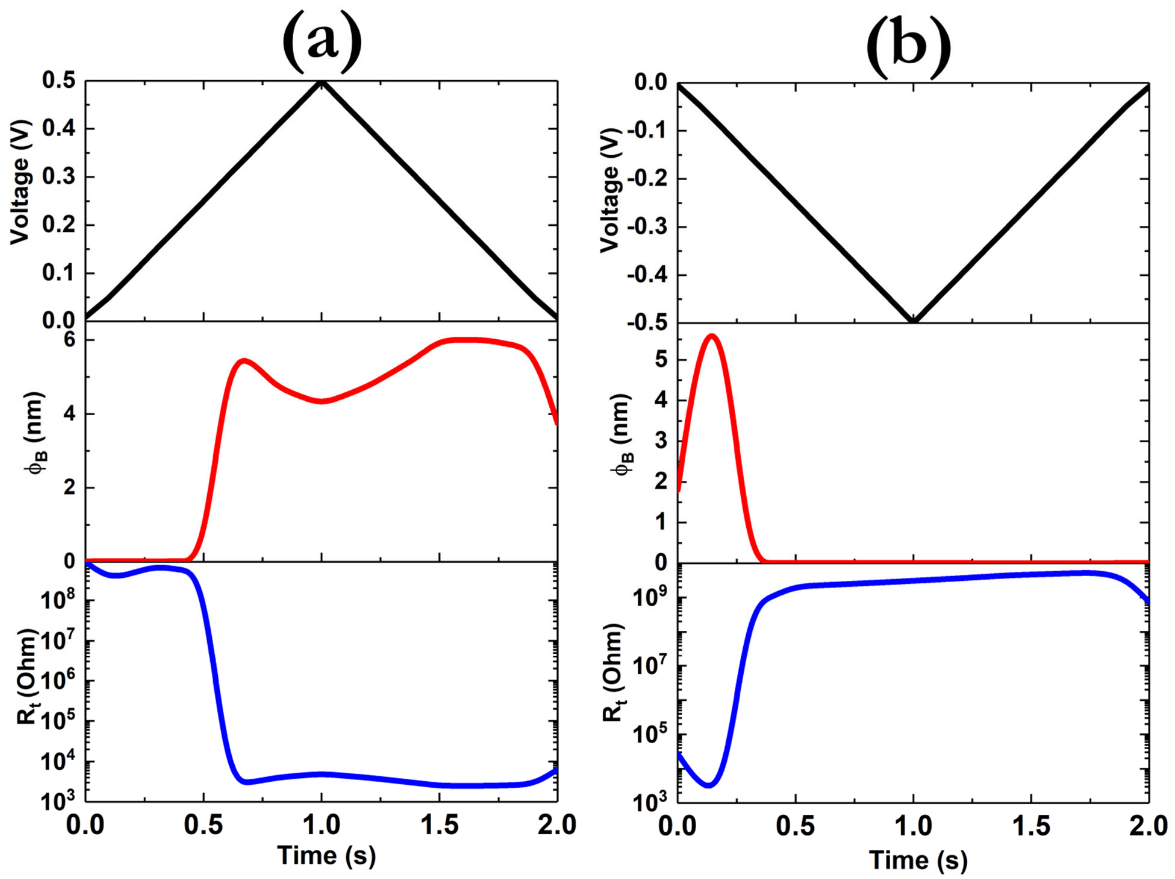

Figure S6. Distribution of the calculated applied bias, bottom diameter of the CF and total resistance during (a) SET and (b) RESET transitions.

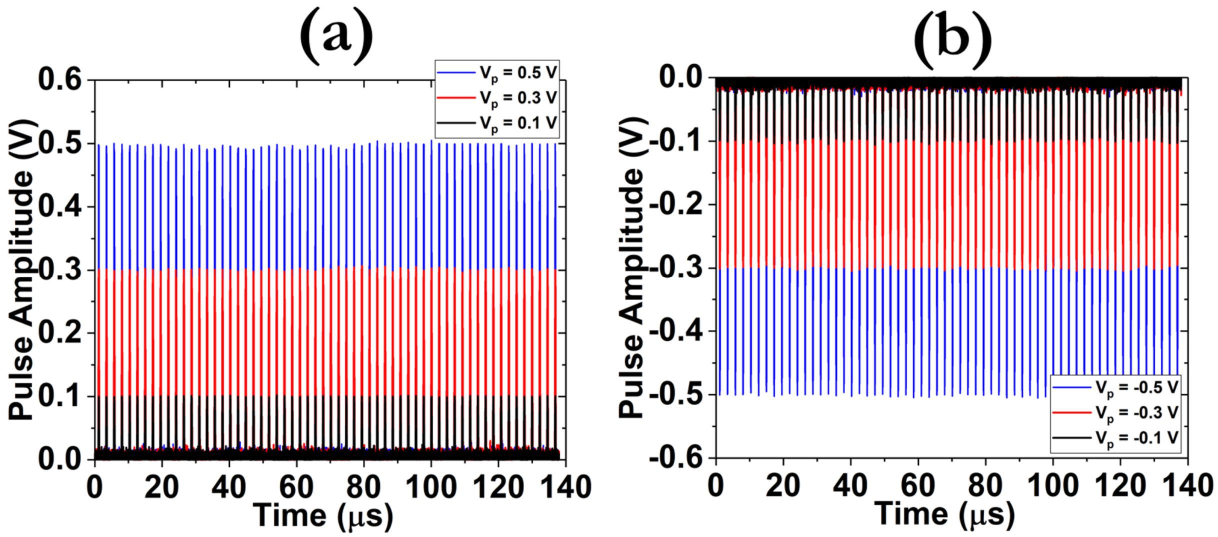

Figure S7. Distribution of the pre-synaptic pulse profiles during the implementation of the (a) potentiation and (b) depression procedures, under the application of total 60 pulses with 100 ns width and amplitudes of ±0.1 V, ±0.3 V and ±0.5 V, respectively.

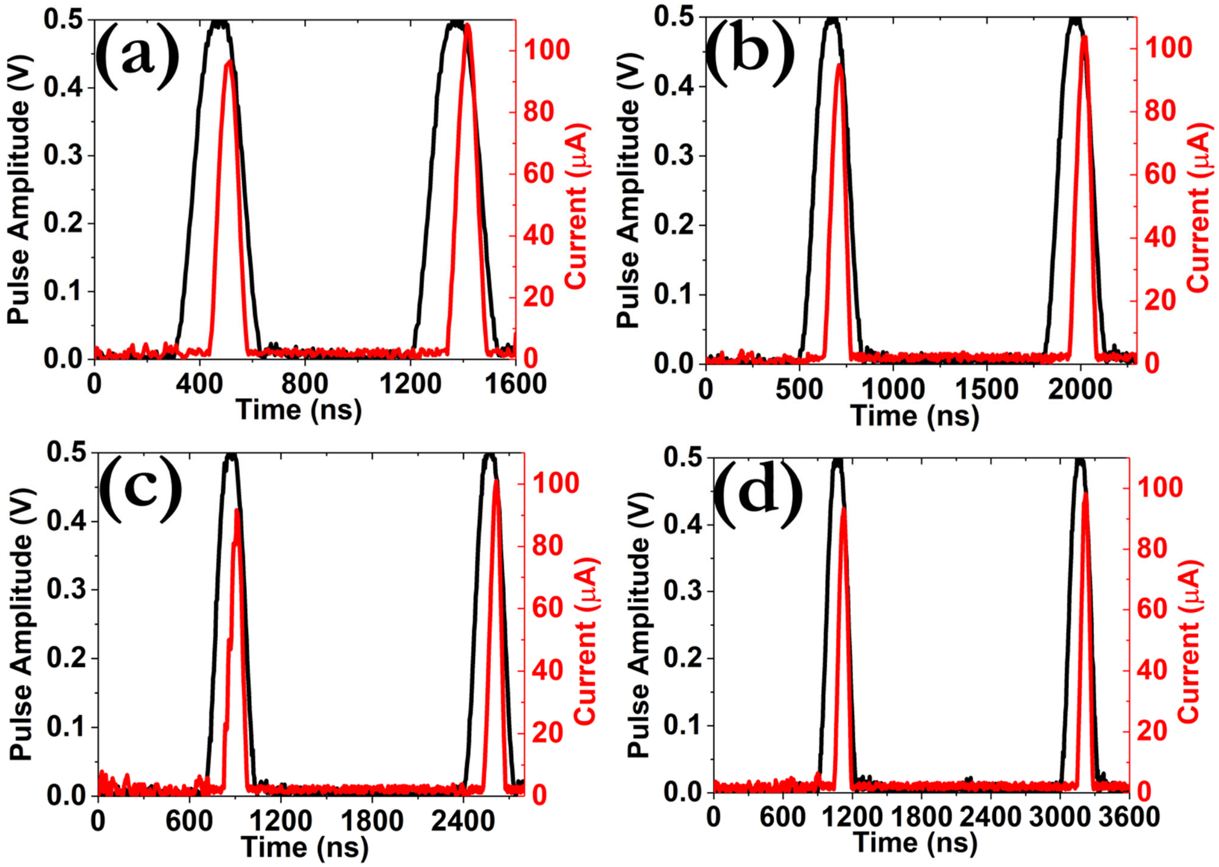

Figure S8. Measured transient current responses (red line) as a function of the pulse delay time (black line) during the enforcement of a set of two square pulses with fixed amplitude (0.5 V), width (100 ns) and delay time of (a) 300 ns, (b) 500 ns, (c) 700 ns and (d) 900 ns, respectively.

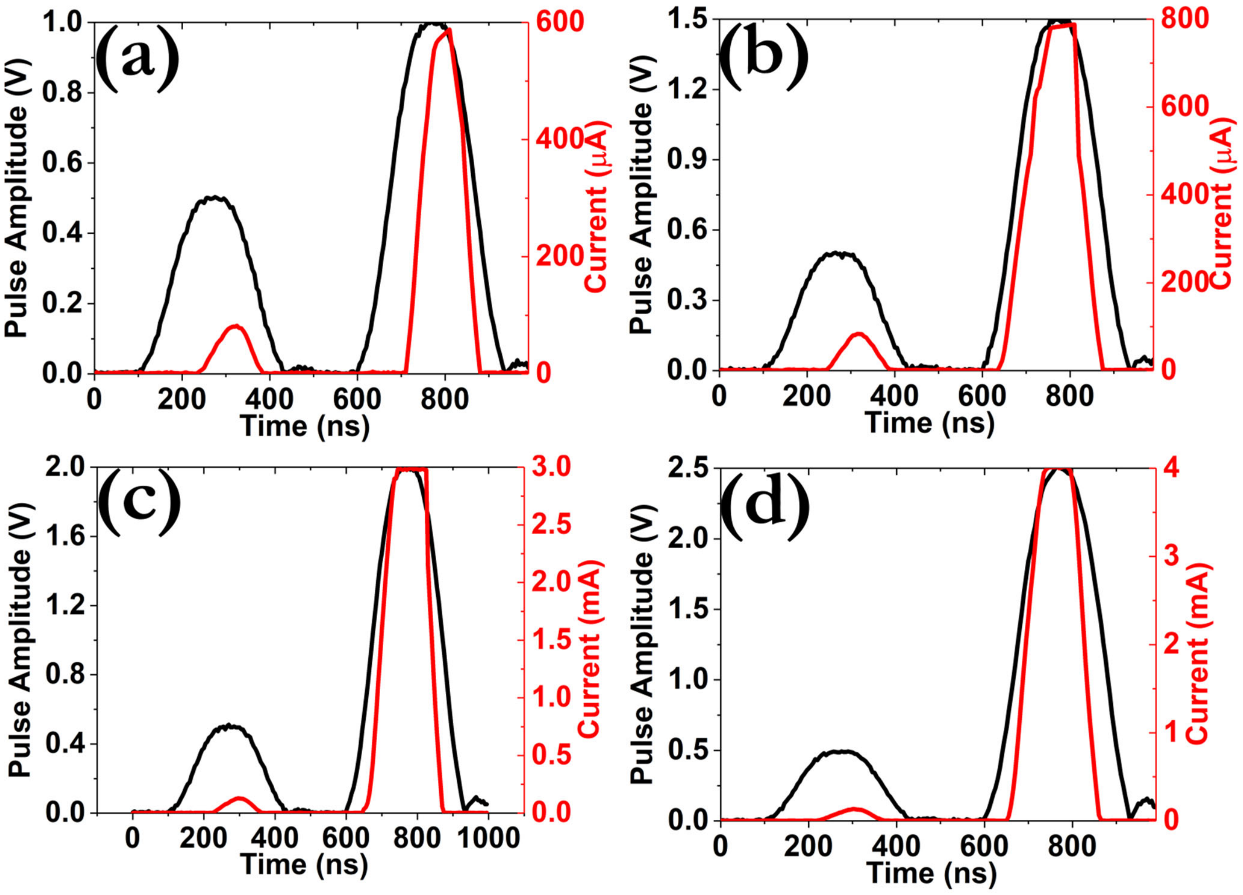

Figure S9. Measured transient current responses (red line) as a function of the pulse amplitude (black line) during the enforcement of a set of two square pulses with fixed delay time (100 ns), width (100 ns) and amplitude of the second pulse of (a) 1 V, (b) 1.5 V, (c) 2 V and (d) 2.5 V, respectively. The amplitude of the first pulse was always kept constant at 0.5 V.
.

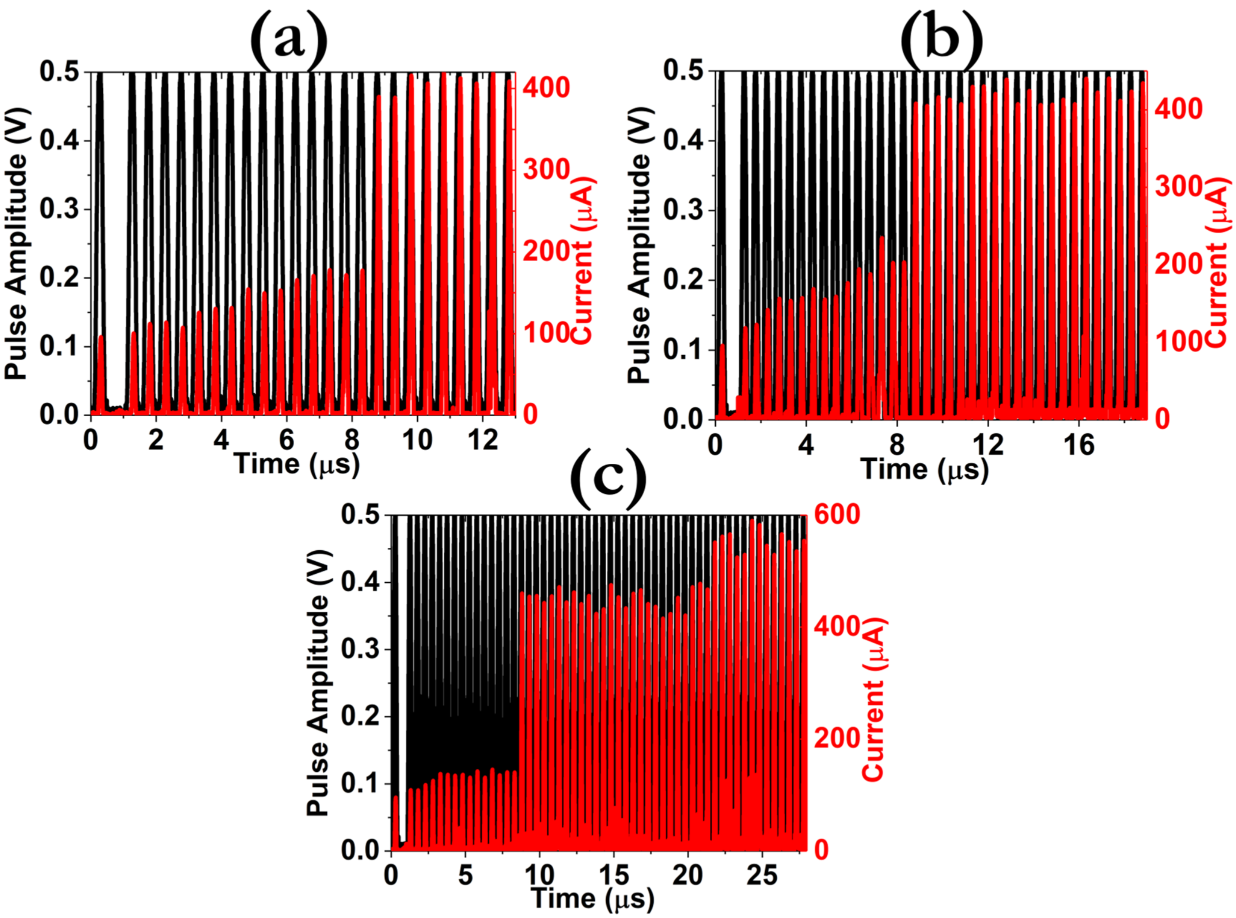

Figure S10. Measured transient current responses (red line) as a function of the pulse number sequence (black line) during the enforcement of a train of square pulses with fixed delay time (100 ns), width (100 ns), amplitude (0.5 V) and a total number of the pulse train of (a) 24, (b) 36 and (c) 54, respectively. The train of the pulses was enforced after the application of a single pulse of 0.5 V amplitude and 100 ns width, while the delay time was set to 1 μs. .

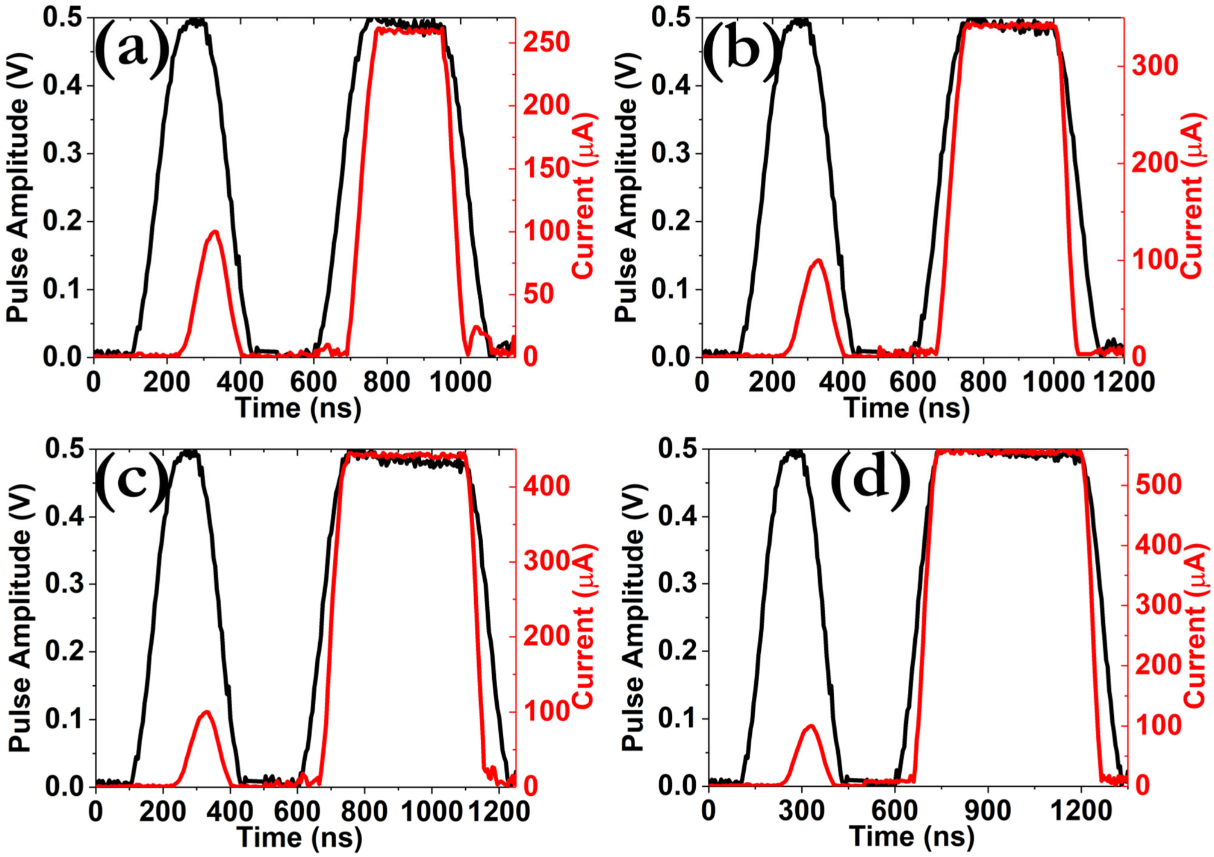

Figure S11. Measured transient current responses (red line) as a function of the pulse width (black line) during the enforcement of a set of two square pulses with fixed delay time (100 ns), amplitude (0.5 V) and width of the second pulse of (a) 250 ns, (b) 300 ns, (c) 400 ns and (d) 500 ns, respectively. The width of the first pulse was always kept constant at 100 ns. .

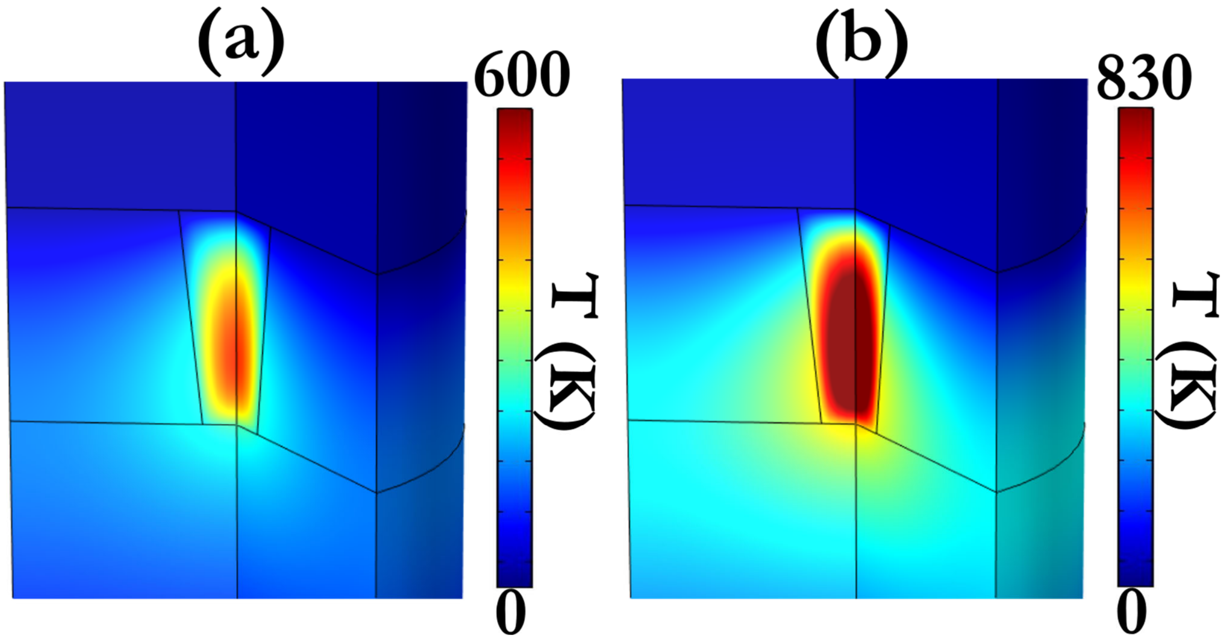

Figure S12. 3D calculated maps of the localized temperature distribution for 40 nm thick TiN BE during (a) SET transition (+0.3 V) and (b) RESET transition (-0.1 V).

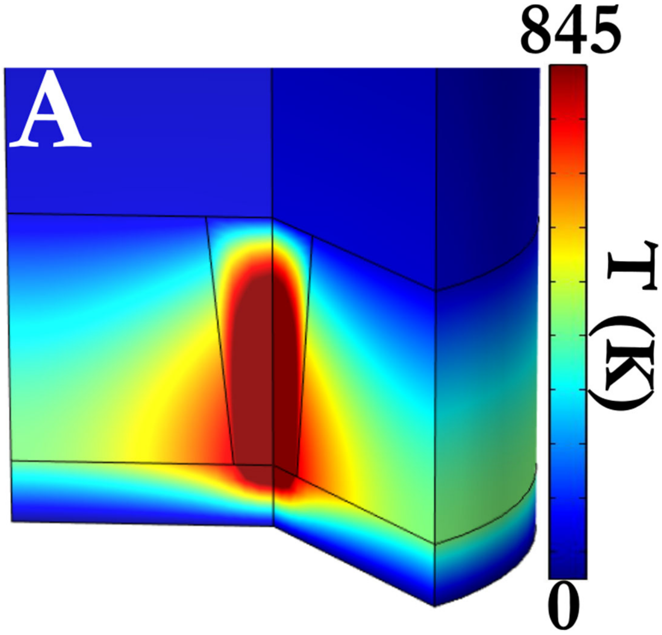

Figure S13. 3D calculated maps of the localized temperature distribution for 5 nm thick Pt NPs BE during SET transition (+0.3 V).

Table S1. Model parameters values.

Parameter	Positive Bias		Negative Bias
E_{drift}		1.1 eV	
E_{diff}		1.3 eV	
E_s		5 eV	
α	0.29		0.84
A	2×10^{-3} ms^{-1}		8×10^{-5} ms^{-1}
B	5×10^{-11} m^2s^{-1}		5×10^{-13} m^2s^{-1}
C	1×10^{-26} m^3s^{-1}		1×10^{-25} m^3s^{-1}
$\rho_{bulk,m}$		Ag: 1.59×10^{-8} Ωm<br>Cu: 1.68×10^{-8} Ωm<br>TiN: 1×10^{-6} Ωm<br>Pt NPs: 2×10^{-7} Ωm	
$\rho_{bulk,SiO2}$		1×10^3 Ωm	
p		0.5	
λ		28 nm	
γ		5×10^{-8} mV^{-1}	
$k_{th,SiO2}$		10 $Wm^{-1}K^{-1}$	
$k_{th,Ag}$		398 $Wm^{-1}K^{-1}$	
$k_{th,TiN}$		1 $Wm^{-1}K^{-1}$	
$k_{th,Pt\ NPs}$		0.1 $Wm^{-1}K^{-1}$	
m		Ag: 5×10^8 $Wm^{-2}K^{-1}$	
ρ_m		Ag: 10.497 kgm^{-3}<br>Pt NPs: 21.447 kgm^{-3}<br>TiN: 5430 kgm^{-3}<br>SiO_2: 2270 kgm^{-3}	
C_p		Ag: 238 $Jkg^{-1}K^{-1}$<br>Pt NPs: 125 $Jkg^{-1}K^{-1}$<br>TiN: 333 $Jkg^{-1}K^{-1}$<br>SiO_2: 133 $Jkg^{-1}K^{-1}$	
